# National Survey to Evaluate the Approach to ACL Injuries among Brazilian Sports Surgeons

**DOI:** 10.1055/s-0045-1812463

**Published:** 2025-11-18

**Authors:** Eduardo Frois Temponi, Lúcio Honório de Carvalho Júnior, Matheus Braga Jacques Gonçalves, Pedro Martins da Costa Drummond, Vitor Rodrigues de Miranda, Waldir de Souza Fernandes Júnior

**Affiliations:** 1 Hospital Madre Teresa, Belo Horizonte, MG, Brazil

**Keywords:** anterior cruciate ligament, anterior cruciate ligament reconstruction, knee, surveys and questionnaires, inquéritos e questionários, joelho, ligamento cruzado anterior, reconstrução do ligamento cruzado anterior

## Abstract

**Objective:**

To describe the profile of Brazilian knee surgeons and their preferences in the diagnosis, treatment, and rehabilitation of anterior cruciate ligament (ACL) injuries, and to compare the findings with data from the current literature.

**Methods:**

We conducted an electronic survey among surgeons who were members of the Brazilian Society of Arthroscopy and Sports Traumatology (Sociedade Brasileira de Artroscopia e Traumatologia Esportiva, SBRATE, in Portuguese). The study included 746 orthopedic surgeons with registered email addresses. We sent a message with an invitation to participate, the research objectives, and a link to access the questionnaire, which contained 36 questions about the professional profile of the surgeons and their preferences in the diagnosis, surgical technique, and rehabilitation of ACL injuries.

**Results:**

The number of participants was 170 (22.7% of the 746 orthopedic surgeons invited). All participants performed ACL reconstruction, with 72% having more than 10 years of experience and 43.5% performing more than 50 reconstructions per year. Sports trauma was the most common injury mechanism (95%). Instability (95.3%) and return to sport (82.2%) were the main criteria for surgical indication. Most participants (53.5%) indicated surgery 1 to 4 weeks after injury. Flexor grafting (88.2%) and the in-out transportal technique (39.2%) were the most frequent options. The predominant postoperative approaches were immediate weight-bearing (51.8%) and early full range of motion (61.3%).

**Conclusion:**

The study outlined the profile and practices of SBRATE knee surgeons in ACL reconstruction. Our findings demonstrate that Brazilian practices follow global trends described in the recent literature.

## Resumo

**Objetivo:**

Descrever o perfil dos cirurgiões de joelho brasileiros e suas preferências no diagnóstico, tratamento e reabilitação da lesão do ligamento cruzado anterior (LCA), e fazer uma comparação com dados da literatura atual.

**Métodos:**

Realizou-se um levantamento eletrônico entre cirurgiões membros da Sociedade Brasileira de Artroscopia e Traumatologia Esportiva (SBRATE). Foram incluídos 746 ortopedistas com endereço cadastrado. Foi enviada mensagem com o convite para participação, os objetivos da pesquisa, e o *link* para acesso ao questionário, que continha 36 perguntas sobre perfil profissional, diagnóstico, técnica cirúrgica e reabilitação da lesão do LCA.

**Resultados:**

O número de participantes foi de 170 (22,7% dos 746 ortopedistas convidados). Todos os participantes realizavam reconstrução do LCA, sendo que 72% tinham mais de 10 anos de experiência, e 43,5% realizavam mais de 50 reconstruções anuais. O trauma esportivo foi o mecanismo de lesão mais comum (95%). Instabilidade (95,3%) e retorno ao esporte (82,2%) foram os principais critérios para indicação cirúrgica. A maioria (53,5%) indicava cirurgia entre a primeira e a quarta semanas após a lesão. Enxerto dos flexores (88,2%) e técnica transportal *in-out* (39,2%) foram as opções mais frequentes. No pós-operatório, carga imediata (51,8%) e arco de movimento completo precoce (61,3%) foram as abordagens predominantes.

**Conclusão:**

O estudo delineou o perfil e as condutas dos cirurgiões de joelho da SBRATE na reconstrução do LCA. Os achados indicam que as práticas no Brasil seguem as tendências globais descritas na literatura recente.

## Introduction

The reconstruction of the anterior cruciate ligament (ACL) has undergone significant advancements throughout the past few decades. The established technique using autologous patellar grafts fixed with interference screws has lost its hegemony in recent studies. 1 2 3 More precise anatomical descriptions, followed by clinical and biomechanical studies, changed current trends in several aspects of treatment. 4 5 The concept of anatomical reconstruction has redefined the goals of ligament restoration, demonstrating better outcomes than isometric reconstructions. 6 7 The literature widely debates topics such as femoral tunnel drilling, type of fixation device, preservation of remnants, maintenance of the tibial insertion of the flexor tendons, and combined extra-articular reconstruction. Although different methods present technical particularities, their proper execution results in comparable efficacy 8 9 10 and a positive impact on quality of life. 8 11

Diagnostic, therapeutic, and prognostic protocols for ACL injuries are constantly updated. Although studies with level I of evidence establish quality standards and guide clinical decisions, patient reports often diverge from physician evaluations, making objective analysis of results difficult. 11 12 Despite extensive scientific production, external factors influence the surgeons' conduct in the daily practice. 1 In Brazil, the disparity between the public and private systems results in economic and social differences that impact therapeutic choices. 13 The increased incidence of ACL injuries, which is linked to new activity patterns, reinforces the importance of understanding the current practices in Brazil. 12 In the United States, an estimated 200 thousand to 250 thousand injuries occur annually, with an approximate cost of 13 thousand dollars per surgery. 14

Survey questionnaires are valuable for collecting data and presenting practices, attitudes, and trends. Researchers use these questionnaires for quantitative data analysis, which plays an essential role in clinical epidemiology and healthcare services. 15 16 A systematic review by Ekhtiari et al. 17 included 20 questionnaires on ACL reconstruction surgery practices in several regions of the world. Brazilian descriptive data on ACL injuries are scarce. Ambra et al. 1 conducted a study during a Brazilian event and reported that surgeons' choice depends on their experience and available resources. The current study aimed to describe the epidemiological profile of ACL reconstruction in Brazil and to compare it with global surveys that address controversial topics in the literature.

## Materials and Methods

The Committee of the Brazilian Society of Arthroscopy and Sports Traumatology (Sociedade Brasileira de Artroscopia e Traumatologia Esportiva, SBRATE, in Portuguese) and the Ethics Committee of our institution approved the study under number CAAE 78506324.6.0000.5127.

The present study involved an online survey applied to orthopedic surgeons specializing in ACL reconstruction who are members of SBRATE. We developed a structured questionnaire on the Google Forms platform (Alphabet Inc.) about the epidemiological profile of ACL reconstructions in Brazil. Before its application, the questionnaire underwent a process of content validation, consisting of its review by a group of surgeons specialized in knee surgery at our institution, to ensure the clarity, relevance, and appropriateness of the questions to the Brazilian clinical context. We emailed an invitation for participation, a consent form, a description of the study's objectives, and a link to access the questionnaire to 746 registered SBRATE members. The first email, on January 5, 2023, was opened by 249 members, 79 of whom clicked on the link. A second email, 14 days later, was opened by 231 members, 41 of whom clicked on the link. The response period remained open until August 5, 2023, to maximize participation. The responses were anonymous, and the system prevented duplication. The study consisted of a detailed questionnaire ( **Appendix 1** ) addressing the surgeons' profiles and preferences and containing 36 questions with 2 potential answer options. In 26 questions, the participants could select one answer, while the remaining 10 questions enabled multiple selections. The initial questions were on the participants' demographic characteristics and professional experience. The following 26 questions addressed diagnostic parameters, approach, and planning. The last 10 questions focused on surgical techniques and aspects of rehabilitation.

### Statistical Analysis

We computed descriptive statistics for each parameter analyzed. We presented the continuous variables as mean values, and the categorical variables, as frequency distributions. We compared our findings with those of recent surveys on global trends in the treatment of ACL injuries.

## Results

### Surgeons' Profile

**Fig. 1:**
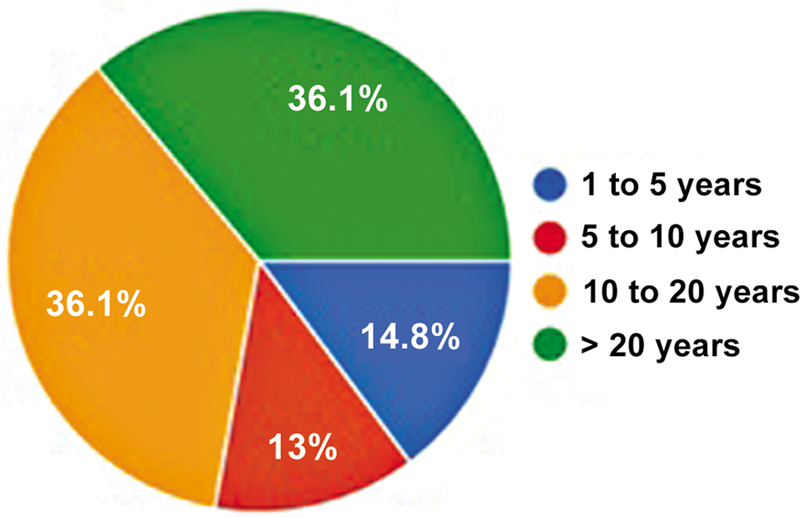
Length of experience with anterior cruciate ligament (ACL) reconstructions.

**Fig. 2:**
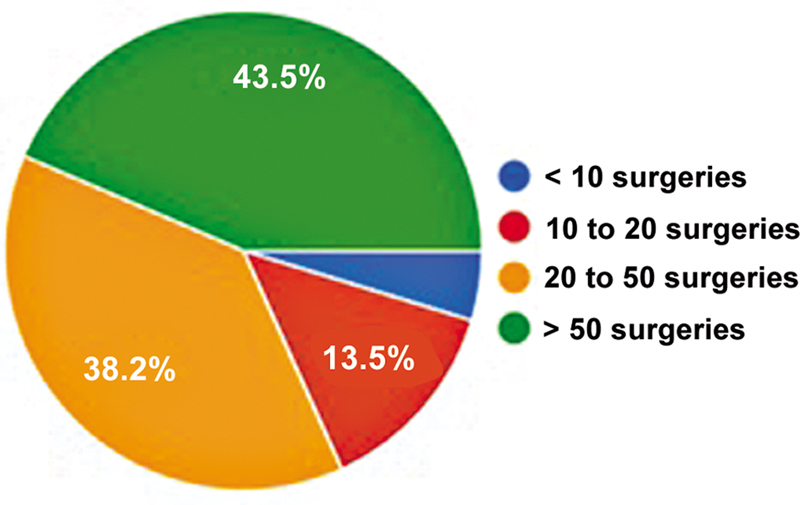
Yearly number of ACL reconstructions.

Of the 746 SBRATE-affiliated orthopedists with registered email addresses, 170 (22.7%) responded to the questionnaire, with only 1.8% being female members. All participants confirmed that they performed ACL reconstruction surgery. The average number of responses per question was of 167.7. The predominant age group was from 40 to 50 years (36.1%), while 14.8% were older than 60 years. Most participants (80%) had been practicing orthopedics for more than 10 years, and 72% had the same length of experience with ACL reconstruction ( Fig. 1 ). Furthermore, 43.5% performed more than 50 surgeries per year ( Fig. 2 ).

### Diagnostic Approach

**Fig. 3:**
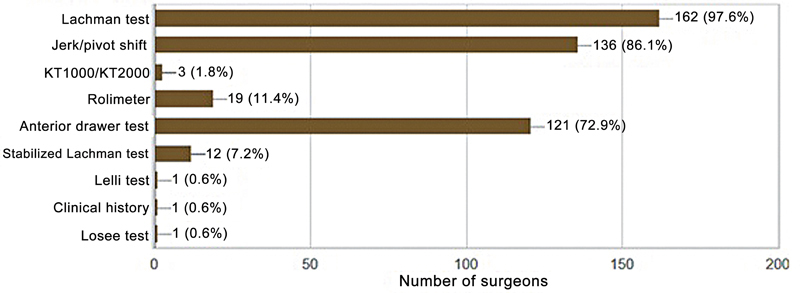
Clinical parameters for diagnosis.

The primary reported cause was sports-related trauma, accounting for 95.9% of the cases. The most common approaches in acute trauma were ice (84.7%) and medication (60.4%). The most frequently used diagnostic methods were clinical examination (93.5%) and magnetic resonance imaging (86%). Regarding magnetic resonance imaging, 85% of participants considered the sagittal section to be the most appropriate for diagnosis, followed by the coronal section (35.2%) and the coronal oblique section (33.9%). The surgeons reported that the Lachman test (97.6%), the pivot shift test (81.9%), and the anterior drawer test (72.9%) were the preferred clinical assessments ( Fig. 3 ).

### Surgical Technique and Rehabilitation

**Fig. 4:**
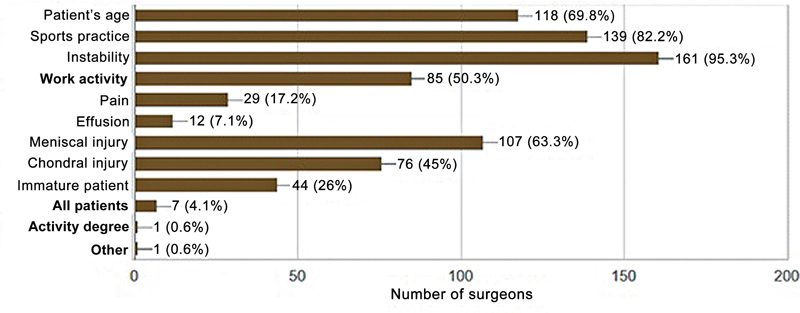
Factors for indication of ACL reconstruction.

**Fig. 5:**
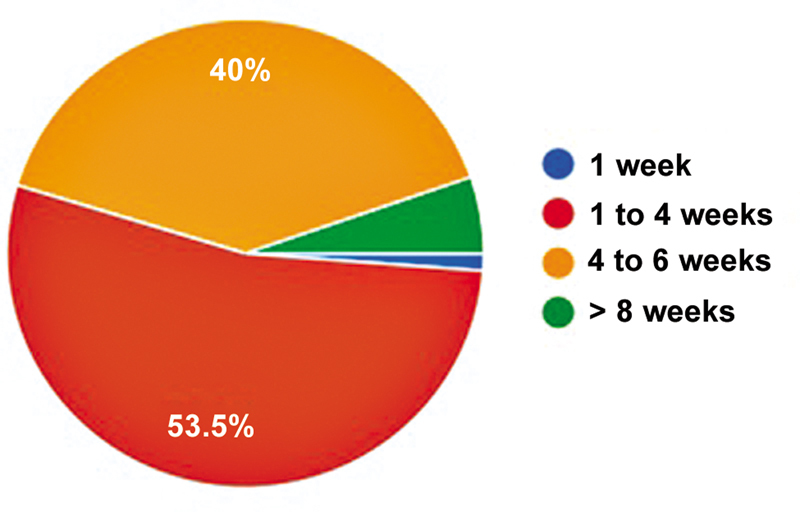
Preferred postinjury time for ACL reconstruction.

Patient-reported knee instability was the main indication for surgical treatment (95.3%), followed by the desire to return to sports. Age and meniscal injury were also parameters taken into consideration for surgical decision-making ( Fig. 4 ). The preferred timing for most surgeons to perform surgery was between 1 and 4 weeks ( Fig. 5 ). Spinal anesthesia (58.6%), use of a tourniquet (98.2%), and surgical time between 30 and 60 minutes (68%) were the most frequently reported practices.

**Fig. 6:**
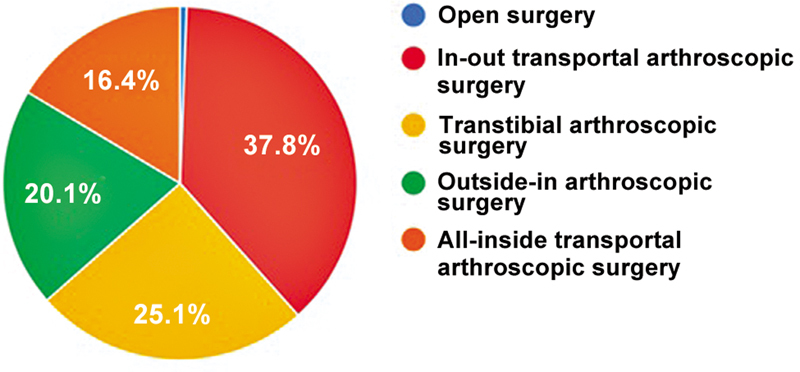
Preferred ACL reconstruction technique.

Most surgeons (88.2%) selected autologous flexor tendon, and 72.6% of them performed pretension. Concerning the femoral tunnel drilling technique, 37.8% of the surgeons used the in-out transportal approach, and 25.1% preferred the transtibial approach ( Fig. 6 ). A little over a quarter of the surgeons (26.7%) reported using the medial accessory portal.

In the presence of ligament remnants, 41% of the surgeons fully preserved the tissue stump, while only 13.3% chose to remove it completely. Most surgeons (47.1%) indicated notchplasty in cases of intercondylar narrowing, and 29.4% performed it in the presence of graft impingement during the range of motion. A small percentage of surgeons (11.6%) mentioned anterolateral ligament (ALL) reconstruction, while 51.8% did not perform the procedure, and 34.1% recommended it only in specific situations. The interference screw was the implant most commonly used for fixation (77.6%), with a preference for the absorbable model; meanwhile, the remaining participants (22.4%) selected suspension fixation devices with an adjustable button.

**Fig. 7:**
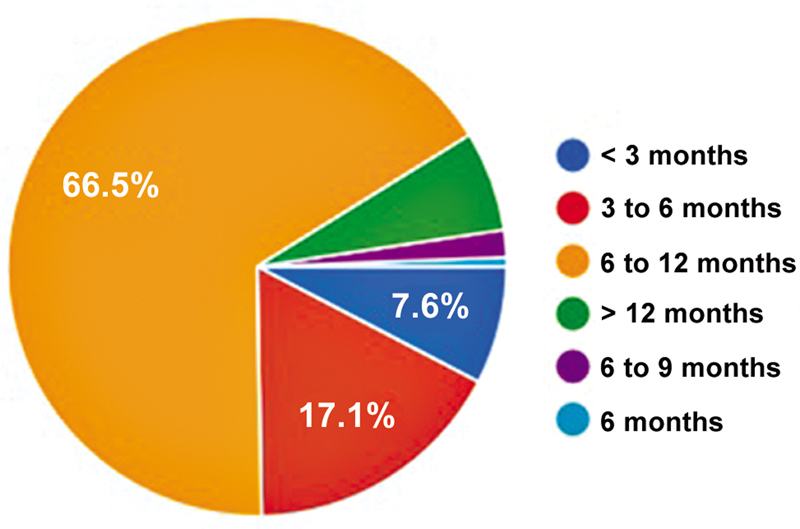
Time to allow physical activities after ACL reconstruction.

**Fig. 8:**
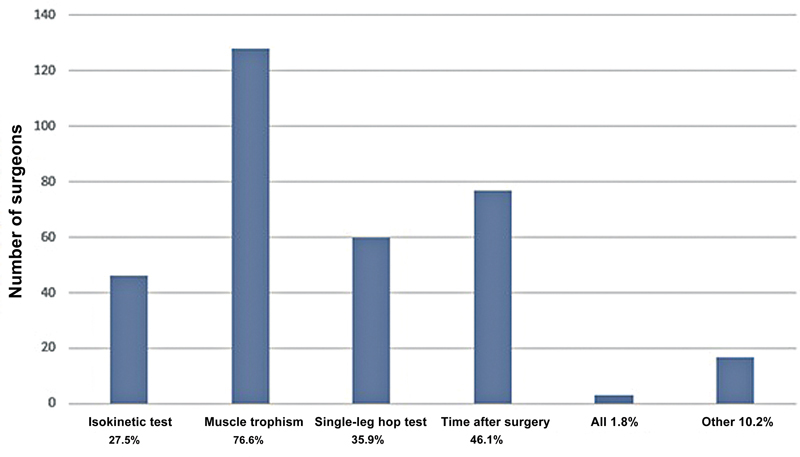
Criteria for returning to sports.

Postoperative physical therapy was routine for 99.4% of the orthopedic surgeons. More than half (51.8%) of the participants reported immediate weight-bearing, and 61.3% did not impose any restrictions on the range of motion. Only 10.2% recommended the postoperative use of braces. Most surgeons (66.5%) allowed physical activity after 6 to 12 months ( Fig. 7 ), and 59.3% reported that this was the length of time expected by the patients. The main criteria for return were muscle trophism (76.6%), postoperative time (46.1%), and the single-leg hop test (35.9%) ( Fig. 8 ).

**Fig. 9:**
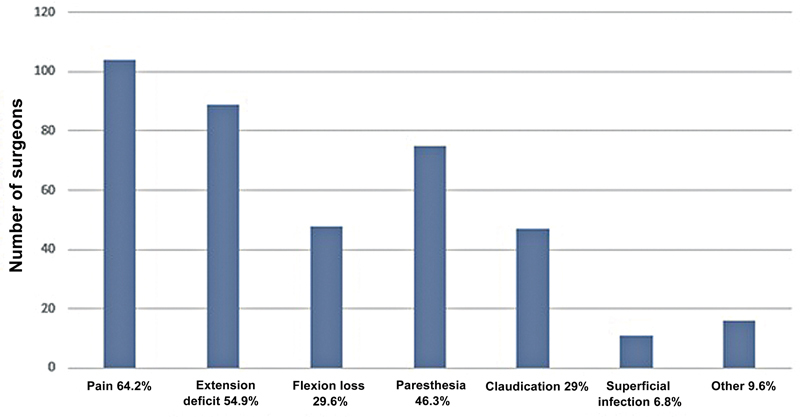
Short-term complications.

**Fig. 10:**
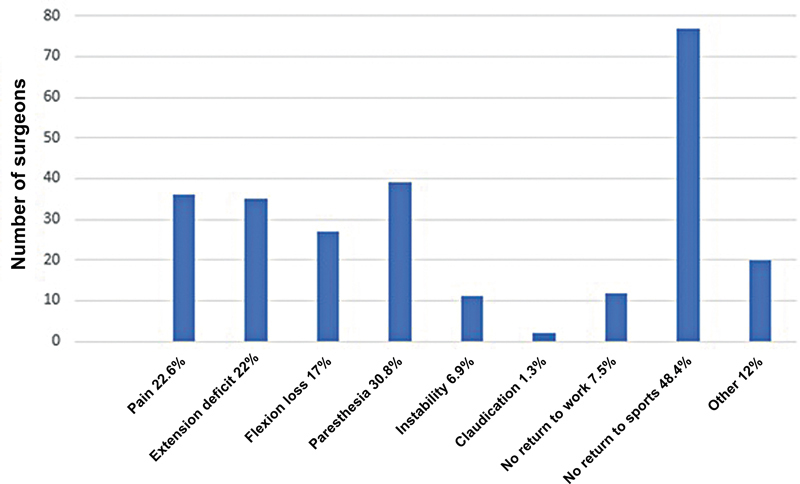
Long-term complications.

The most frequently reported short-term complications were pain (64.2%), extension deficit (54.9%), paresthesia (46.3%), loss of flexion (29.6%), claudication (29%), and superficial infection (6.8%) ( Fig. 9 ). After 6 months, the main complication was non-return to physical activities (48.4%), followed by paresthesia (30.8%) ( Fig. 10 ). There was a reduction in the frequency of pain, extension deficit, loss of flexion, and claudication during this period.

## Discussion

The survey aimed to investigate the profiles and preferences of surgeons specializing in ACL reconstruction working in Brazil. Although the literature describes similar surveys among society members in several parts of the world, 1 2 12 18 19 the current study is the first Brazilian national survey to map the trends of 170 surgeons with expertise in ACL reconstruction. Among the participants, 72% had been performing reconstructions for more than 10 years, and 43% performed more than 50 procedures annually. In the United States, Erickson et al. 20 surveyed experienced surgeons from the National Football League (NFL) teams in 2014; they reported an average of 82 reconstructions per year and an average experience of 16.8 years. Shafizadeh et al. 3 found an average of 35 surgeries per year, differentiating high-volume (more than 50 reconstructions per year) from low-volume surgeons. The results showed significant differences between them regarding association membership, procedure duration, use of stability tests, and preferred drilling technique. The parameters most frequently cited to indicate reconstruction were subjective instability and the Lachman test. Few reported using stability measurement instruments, with 17% mentioning the Rolimeter (Aircast Europa), and 10%, the KT1000 (MEDmetric Corporation). Most surgeons preferred a reconstruction period of between 6 weeks and 3 months.

In the current study, the primary factors for surgical recommendation were instability (95%) and participation in sports (82%). Additionally, 93% of the specialists preferred to perform surgery between the first and sixth weeks after the injury. Farber et al. 21 surveyed physicians from Major League Soccer (MLS) teams in the United States. These authors reported that 48% of the surgeons would perform reconstruction within the first 2 weeks, and 33%, between the second and fourth weeks. McRae et al. 18 sought to establish trends through the level of agreement among the responses of Canadian surgeons. Knee instability in daily and physical activities, high demand, and repairable meniscal injury reached rates of agreement higher than 80% as criteria for treatment indication. A donor site ipsilateral to the injury, a single-incision technique, and pretension also reached these levels of agreement.

The preference for autologous flexor grafts was high (88.2%) in the present study. However, the literature still debates which type of graft is best. 22 A systematic review by Reinhardt et al. 23 compared reconstructions using patellar and flexor grafts and showed a higher failure rate in flexor grafts (15.8% versus 7.2%) and more anterior pain with patellar grafts. A previous Brazilian survey 1 found an even higher rate, with 93% of the surgeons opting for flexor grafts. Chechik et al. 24 reported regional variations, as 72% of European and 42% of North-American surgeons preferred flexor grafts. Farber et al. 21 found that 68% of surgeons used autologous patellar grafts in MLS players. Erickson et al. 20 reported that 86% of surgeons preferred patellar tendons in NFL athletes, and 50% selected them for recreational athletes over the age of 25. Cerciello et al. 25 conducted a new survey among young European surgeons and found that the quadriceps tendon is becoming increasingly popular among practitioners in contact sports. These authors reported that young European surgeons prefer using semitendinosus and gracilis tendon grafts for patients with lower demands.

Our findings confirm the consolidation of independent femoral tunnel drilling in Brazil, which is in line with the global trend. Although the literature has not yet demonstrated clinical superiority, 26 only 16.3% of surgeons still use the transtibial technique, a percentage lower than that of the previous Brazilian survey, 1 in which 26.5% preferred it, making this finding even more relevant. No surgeon with more than 15 years of experience opted for the anteromedial transportal technique. In international studies, 24 68% of the specialists prefer the anteromedial transportal approach, and 31% select the transtibial approach. Erickson et al. 20 reported 67% of preference for drilling through the accessory medial portal and 25% for the transtibial approach. Farber et al. 21 found that 50% of surgeons still use the transtibial technique. Another relevant change identified was the greater preservation of the ligament remnant. Only 13.3% of the participants still perform complete stump removal. In 2011, McRae et al. 18 found a correlation between its sparing and the highest volume of reconstructions in Canada; however, among all participants, 45% still sacrificed the remnant in full.

Reconstruction of the ACL combined with a lateral extra-articular procedure may improve knee rotational stability control compared with reconstruction alone. However, its indication remains controversial due to uncertainties regarding the actual stability gain and the ideal patient profile. Currently, surgeons prefer ALL reconstruction in cases of high-grade pivot shift and procedural revisions. 27 Tramer et al. 28 reported that 59.6% of surgeons do not perform ACL and ALL reconstructions together. This finding is consistent with that of our study, in which 51.8% of the respondents did not adopt this technique.

The rehabilitation results show that Brazilian surgeons adopt accelerated protocols, with early weight-bearing and full range of motion, a trend consistent with 77% of the participants in the study by Campbell. 29 McRae et al. 18 observed a similar result, with 72% of the surgeons allowing full weight-bearing and 75% allowing full range of motion immediately after surgery. A survey of 46 American surgeons by Glattke et al. 30 suggested that a high percentage of them use postoperative orthoses for a period ranging from 3 to 6 weeks. However, this topic still presents considerable variability among the protocols adopted by orthopedic surgeons.

A survey conducted by Petersen and Zantop 19 listed criteria for returning to competitive sports, with 63.5% of surgeons recommending a return after 6 months and 76.6% beginning specific exercises within 4 months, based primarily on a negative Lachman test and full range of motion. Other parameters included proprioceptive testing (43%), the single-leg hop test (39%), and muscle strength (40%). However, 85.8% of the study participants did not follow specific parameters. In the current study, more than 2/3 of the Brazilian surgeons recommended a return between 6 and 12 months, which is consistent with the study by Vascellari et al., 2 who also identified a recommendation of return between 6 and 8 months in 73% of cases. At 4 weeks, 33% of the surgeons allowed stationary cycling, and only 16% allowed jogging after 8 weeks. Erickson et al. 20 found that 74.5% of the surgeons allowed return to sport in NFL athletes based on the single-leg hop test, 55.4% suggested a minimum time of 6 months after surgery, and 56% required a normal range of motion, absence of pain, regular strength, and stability. 20 A systematic review by Mayer et al. 31 highlighted the importance of functional tests for a safe return to sport, although there is no consensus on which tests are the most effective.

A study by Grassi et al. 32 evaluated recommendations for return to sport after ACL reconstruction. Most participants (87%) cited a 6-month timeframe to return to non-contact sports, and 43%, for contact sports. At the competitive level, return rates within 6 months dropped to 48% for non-contact sports and to 13% for contact sports. There was no significant difference regarding graft type or surgical experience. The criteria most frequently used were the total range of motion, the Lachman test, and the pivot shift test, with 90% of the surgeons adopting at least one of them. Only half of the respondents used instrumental assessment of muscle strength, and 30% applied functional tests. Only 10% of the participants recommended braces, contrasting with the 49% rate reported by Vascellari et al. 2 Farber et al. 21 observed a higher frequency of brace use in reconstructions using flexor grafts. According to Mayer et al., 31 the lack of standardization in rehabilitation protocols and return-to-sport testing is an ongoing challenge, and more high-quality research is needed to establish evidence-based recommendations.

The current study was the first online survey conducted with surgeons specializing in the treatment of ACL injuries in Brazil. The qualifications of the participants ensured the reliability of the data. The main limitation was the low response rate (22.7%). Similar studies in Italy, such as those by Grassi et al. 32 and Vascellari et al., 2 found participation rates of 16% and 17%, respectively. Other international surveys had fewer than 50 participants in their sample. 13 21 Erickson et al. 20 recorded a 51% participation rate among 267 referral surgeons for NFL teams. The systematic review by Ekhtiari et al. 17 mentioned methodological aspects related to the survey's success in their investigation on the quality of surveys on ACL reconstruction. Their systematic review included 35 studies, with an average response rate of 73%. These authors concluded that adherence varies according to the audience and distribution method, being higher in in-person and patient-directed surveys.

## Conclusion

The present study described the profile and preferences of SBRATE member knee surgeons who perform ACL reconstruction. Knee instability was the main indication for surgical treatment. Furthermore, autologous flexor tendon grafting was the preferred choice among participants, and muscle trophism was the most cited parameter for returning to physical activity. Our results show that Brazilian surgeons seem to incorporate the global trends described in recent surveys.

## Notes

Appendix 1 Survey questionnaire

1) What is your gender?
  Male; female.
2) How old are you?
  20 to 30 years; 30 to 40 years; 40 to 50 years; 50 to 60 years; > 60 years.
3) How long have you been practicing orthopedics?
  1 to 5 years; 5 to 10 years; 10 to 20 years; > 20 years.
4) Do you perform anterior cruciate ligament (ACL) reconstruction?
  Yes; no.
5) How long have you been performing ACL reconstruction?
  1 to 5 years; 5 to 10 years; 10 to 20 years; > 20 years.
6) How many anterior cruciate ligament reconstruction surgeries do you perform per year?
  < 10 surgeries; 10 to 20 surgeries; 20 to 50 surgeries; > 50 surgeries.
7) What do you prefer after an ACL trauma?
  -Ice; support/weight-bearing restriction; early mobilization; medication; physical therapy.
8) What is the main cause of ACL injury in your clinical practice?
  - Sports trauma; trauma due to road traffic accident; low-energy trauma (falls); other.
9) What diagnostic method for ACL injury do you use in your clinical practice?
  Clinical examination: magnetic resonance imaging; magnetic resonance arthrography; diagnostic arthroscopy.
10) What clinical parameters do you use to diagnose an ACL injury?
  Lachman test; jerk/pivot shift test; KT 1000/KT 2000; Rolimeter; anterior drawer test; stabilized Lachman test.
11) In the magnetic resonance imaging evaluation, which section do you use to diagnose an anterior cruciate ligament injury?
  Sagittal; coronal; oblique-coronal; axial; others.
12) What factors do you consider when indicating an ACL reconstruction?
  Patient's age; sports practice; instability; patient's occupation; pain; effusion; associated meniscal injury; associated chondral injury; immature patient; I perform reconstruction in all patients.
13) After an ACL injury, what is your preferred time for reconstruction?
  1 week; 1 to 4 weeks; 4 to 6 weeks; > 8 weeks.
14) Which anesthesia do you select, together with the anesthesiology team, for the surgical procedure?
  Spinal anesthesia; spinal anesthesia + peripheral block; epidural; general anesthesia; general anesthesia + peripheral block.
15) Do you use a tourniquet during surgery?
  Yes; no.
16) On average, in an isolated injury, how long is the operative time for ACL reconstruction?
  < 30 minutes; 30 to 60 minutes; 60 to 90 minutes; > 90 minutes.
17) Which graft do you prefer to use for primary ACL reconstruction?
  Patellar tendon; flexor tendons; quadriceps tendon; fibular tendon; allograft; contralateral flexor tendons.
18) Which technique do you prefer for ACL reconstruction?
  Open surgery; in-out transportal arthroscopic surgery; in-out transtibial arthroscopic surgery; outside-in femoral arthroscopic surgery; all-inside arthroscopic surgery; transtibial all-inside arthroscopic surgery
19) Which portals do you routinely use during the arthroscopic reconstruction procedure?
  Anterolateral and anteromedial; anterolateral, anteromedial, and accessory medial.
20) In the presence of ACL remnants, what is your choice during reconstruction?
  I fully sacrifice the remnants; I partially sacrifice the remnants; I fully keep the remnants.
21) Do you perform notchplasty during the surgical procedure?
  Always; never; when the intercondyle is narrow; when there is graft impingement on the intercondyle wall.
22) Which graft fixation method do you prefer?
  Interference screw; Endobutton; cross pin; post; others
23) What type of implant material do you prefer?
  Metallic; absorbable; polyether ether ketone (PEEK); others.
24) Do you pretension the graft during the fixation process?
  Yes; no.
25) Do you use a drain after the surgical procedure?
  Yes; no.
26) Do you perform anterolateral ligament reconstruction?
  Yes; no; sometimes (in specific situations); other.
27) Do you indicate postoperative physical therapy rehabilitation?
  Yes; no.
28) When do you allow weight-bearing after ACL reconstruction?
  Immediately; 1 week; 2 weeks; 4 weeks.
29) When do you allow a full range of motion after the surgery?
  Immediately; 1 week; 2 weeks; 4 weeks or more.
30) Do you recommend the use of an orthosis or some type of immobilization (such as a cast) postoperatively?
  Yes; no.
31) What short-term complications (< 8 weeks) do you encounter in your daily practice?
  Pain; extension deficit; loss of flexion; superficial infection; deep infection; paresthesia; claudication; others.
32) When do you allow physical activity after ACL reconstruction?
  < 3 months; 3 to 6 months; 6 to 12 months; > 12 months.
33) What is the usual time until the return to sports regarding your patients' expectations?
  It is equal to what the patients expect; it is longer than what the patients expect; it is shorter than what the patients expect.
34) What parameter do you use to allow returning to sports practice?
  Isokinetic test; muscle trophism; single-leg hop test; postoperative time; others.
35) Do you recommend the use of an orthosis for sports participation after ACL reconstruction?
  Yes; no.
36) What long-term complications (> 8 months) do you encounter in your daily practice?
  Pain; extension deficit; loss of flexion; paresthesia; instability; claudication; no return to work; no return to sports; others.
